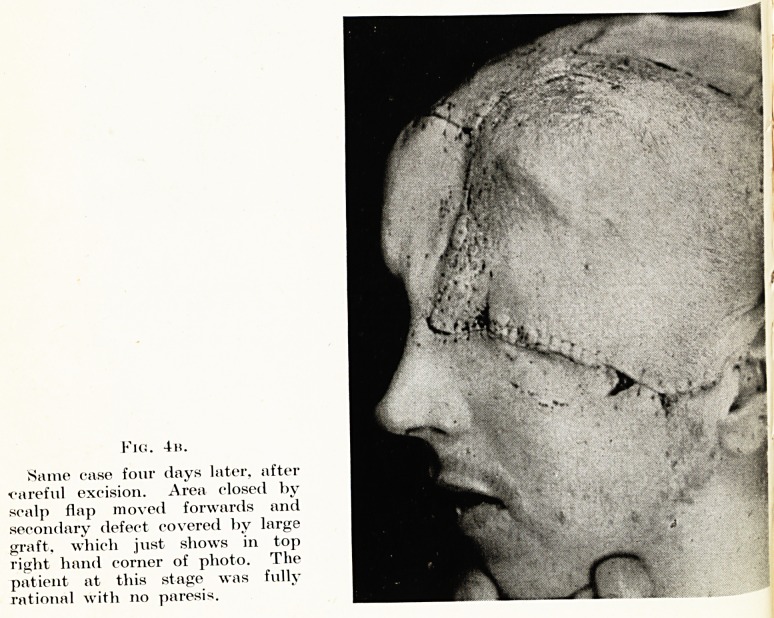# War Injuries of the Face and Jaws
*Summary of a paper read to the Society on Wednesday, October 9th, 1947.


**Published:** 1947

**Authors:** G. M. FitzGibbon

**Affiliations:** Surgeon, Bristol Royal Hospital; Late Lieut.-Colonel, R.A.M.C.


### The Bristol Medico-Chirurgical Journal

*"Scire est nescire, nisi id me*
*Scire alius sciret."*

AUTUMN, 1947.

### WAR INJURIES OF THE FACE AND JAWS.*

BY

G. M. FitzGibbon, M.D., F.R.C.S.

*Surgeon, Bristol Royal Hospital;*
*Late Lieut.-Colonel, R.A.M.C.*

At the outbreak of the war, the War Office decided to create specialist units for the treatment and management of certain types of battle injuries. Amongst these was established a number of maxillo-facial surgical units whose primary function was to deal with injuries of the face and jaws.

The personnel were trained for these units in the main specialist E.M.S. Units of a similar type in this country. The training was an excellent one, and selection was based on the knowledge that general surgery would also have to be undertaken; in fact, rather more than one-third of the casualties with facial injuries had other associated injuries. Facial injuries and burns, i.e. the type of cases which are sent to the maxillo-facial unit, constituted approximately seven per cent. of the total casualties. This figure varied a little with climatic conditions, thus the incidence of burns in the North African campaign was higher than in North-west Europe. In the course of the eleven months active service some 3,000 casualties were admitted to our unit and of these, just over 1,900 needed operations. This represents a lot of work, as these cases are time-consuming, their treatment is elaborate, and often difficult.

* Summary of a paper read to the Society on Wednesday, October 9th, 1947.

Each maxillo-facial unit had its specialist surgeon, dental surgeon, anæsthetist, theatre personnel, and dental mechanics all specially trained. Some months before "D" Day, my unit was posted as a complete unit, to work with an E.M.S. maxillo-facial centre. We had several months of valuable experience, working together as a team, and later experience made one realize how very important this period of preliminary work had been. It enabled the unit to start work smoothly and quickly, as a team, each member knowing what his particular job was, and one had by that time got a very good practical knowledge of what each member of the team was worth.

In order to make the best use of a highly-trained specialist team, and for them to obtain their cases as early as possible, such a unit needs to be placed well forward in the battle area, but not so far forward that cases by-pass it. In other words, the convergence of the various lines of communication covering a large sector of the front is the ideal site. Maxillo-facial cases travel badly, and unless positioned very carefully, will be found on arrival to be partly asphyxiated, with blood and other foreign material in the trachea and lungs. It is important, therefore, that these cases should be dealt with as soon as possible after being wounded. Small specialist units of this type are not self-supporting, and are always attached to a "parent" hospital, who are responsible for feeding, paying, housing, supplying beds, nursing staff, etc. We found that some "parents" were much better than others, and the amount of co-operation one obtained varied in different units. One big oversight was a lack of provision of specially-trained nursing staff to handle these cases. Good nursing by properly-trained personnel makes an enormous difference to the patients' subsequent progress, for inexperienced nurses cannot realize the very grave dangers which may beset a patient coming round from an anæsthetic with his jaws fastened together. The post-operative management was a source of continual trouble which could have been enormously reduced by the provision of a few specially-trained nurses to work with the unit.

The equipment issued to these units was really excellent; it had been well thought out, and apart from one or two deficiencies proved to be adequate. One found that certain things one would have liked were not available, but in a short time, by legal or illegal means, begging, borrowing, scrounging, or otherwise obtaining, we had most of the things we really needed. The biggest difficulty was the replacement and supplies question. A number of the items on the scale of equipment with which we were issued, and to which we were entitled, were not on the scale of issue to Base Depot Medical Stores, and considerable delay occurred before some of our more highly specialized items could be replaced.

### Clinical Problems

*Shock.* It was a constant source of surprise to me, and, I think, to most of my unit, that men with major injuries of the face and jaws, who would appear to be in acute discomfort, were able to sleep for long periods without any apparent effort, and without sedatives. The explanation of this is, I think, two-fold. In the first place, many of the men were seriously short of sleep, owing to having been in action for some time; and secondly—and, I think, a rather more important factor—they all suffered in some measure from concussion. The impact of a high-velocity missile against the bones of the skull must transmit a good deal of violence to the brain. Many of the men were vague and confused in their statements, and fortunately suffered from a considerable amount of amnesia for the events following upon being wounded. There was one very common clinical feature which was observed, in a large number of cases, and tended to confirm the suggestion that considerable violence is transmitted to the whole skull. If one asked a man who was lying down to sit up, any time within three or four days of being wounded, many of them put one hand behind their head so as to give it a support as they raised their body. When one told them to lie down again, they again raised a hand to support the back of the head. They all seemed to suffer from what a casual observer would call a "stiff neck." I suggest that probably there is some synovitis of the joints of the cervical spine, following upon the jarring, concussional effects of the missile striking a bony part of the skull. This sort of stiff neck seemed to pass off after a few days, and with it came a great improvement in general cerebration.

The degree of shock manifested varied a lot, and in the absence of other major injuries responded very well to transfusions. I feel that one should place on record the great debt that is owed to the Army Blood Transfusion Services. They were first-class, and were directly responsible for saving countless lives.

*Asphyxia.* There is a large asphyxial factor responsible for bringing about the deterioration which one saw so often in association with jaw injuries. The cough reflex appears in many instances to be grossly impaired, and patients will tolerate blood clot and mucus in the trachea and lungs which normally they would cough out. This depression of the cough reflex, associated with the fact that they are unable to swallow without great pain and difficulty, nearly always results in a considerable fouling of the trachea and bronchi with blood clot. In addition, in some instances, there was great swelling of the back of the tongue and all the area round the glottic opening, which further added to their embarrassment. Such patients usually had a soft pulse, with a low blood-pressure, and were a peculiar earthy colour, owing to their degree of anoxæmia.

One fact which we very soon learned was that patients who had been subjected to some hours of partial anoxæmia will not stand much operating. It became, after a while, a routine practice merely to do a bronchoscopy, clear the lungs, ensure a good airway, and carry on with resuscitation for sometimes as long as twenty-four hours. These cases would then tolerate a three-hour operation without causing anxiety. In some instances, where it was impossible to establish a satisfactory airway, a temporary tracheotomy was carried out.

It is important to appreciate that certain types of wounds are more dangerous than others in producing asphyxia. A typical instance of the most dangerous type of injury is illustrated in Fig. 1. This man sustained a small puncture wound in the lower right pre-molar region, and the exit was situated below the left ear. The mandible was shattered at the point of entry, and the left angle was also comminuted. The missile, in its passage, severed the left lingual artery, and it can be seen from the photograph that his mouth is completely full of tongue, which is jammed up against the roof of his mouth, and he has a "bull" neck, due to an enormous hæmatoma in the floor of his mouth. This man's condition was so urgent that a tracheotomy was performed in a forward unit, before he was considered safe to evacuate. This case, I believe, illustrates the typical wound produced by a true-running smooth missile. It is particularly dangerous because the entry and exit wounds are so small that there is no drainage possible, and the maximum amount of swelling and dyspnœa results. The type of wound illustrated in Fig. 2 is, I believe, caused by an irregular-shaped missile, such as a piece of shell-casing or a spent bullet which is not running true. It is a horrible and dreadful type of explosive injury, but it does not constitute anything like the same danger to life. The tearing and disruption of the tissues appears to seal off many of the vessels, and the wound is so widely opened that drainage is free, and asphyxial difficulties are not so likely to arise. Although this injury appears at first glance to be a very dangerous one, it is, in fact, less dangerous to life than the less destructive, almost closed type of wound.

*Hæmorrhage.* It is probable that a number of cases died within a few minutes of being hit, from severe primary hæmorrhage, but severe active bleeding was a comparatively rare thing in the cases that survived to reach us. Secondary hæmorrhage was, in our experience, also comparatively rare, and I only tied the external carotid artery on one occasion for secondary hæmorrhage—but one obvious criticism of this statement is that we were not able to hold our cases sufficiently long for this complication to arise. I have been told by surgeons of the plastic surgery centres of this country that secondary hæmorrhage was rare, and I think it can be partly attributed to early and adequate surgery with good drainage, and to the plentiful use of penicillin, and other forms of chemo-therapy.

PLATE III

Fig. 1.

Wound of entry in right mental region, and similar type of exit-wound below left ear. Notice size of tongue, swelling of sub-mental and hyoid region. A tracheotomy tube has been inserted.

### PLATE IV

FIG. 2A.

Massive destructive wound with free drainage and almost no bleeding. Mandible lost from angle to angle.

FIG. 2B.

Same case after closure of mucous membrane to skin. Tongue nearly always escapes major damage. This photo does not give a real impression of the bone lost. A Ryle's tube is in place through the left nostril for feeding, and a temporary tracheotomy has been done, because of the instability of the tongue.

PLATE V

Fig. 3.

Case of high maxillary fracturing, and maxillary block has now been stabilized by a cap splint to a head cap. This photograph was taken after recovery from meningitis, and after operation by Major J. M. Small, for fascial graft to seal off the profuse cerebro-spinal leak through the nasal wound.

PLATE VI

Fig. 4a.

Severe destructive wound involving left orbit and left frontal lobe. *Note.* Left superficial temporal vessels are intact.

Fig. 4b.

Same case four days later, after careful excision. Area closed by scalp flap moved forwards and secondary defect covered by large graft, which just shows in top right hand corner of photo. The patient at this stage was fully rational with no paresis.

*Associated Injuries.* As I have already said, rather more than one-third of all our cases suffered from associated injuries in addition to those to the face. They were, of course, a very big added factor in producing shock, and they constituted a big extra load of work for us to cope with. They produced the usual appearances that many other writers have described, and were treated along conventional standard lines.

### Operative Procedures

Under conditions which obtain in a theatre of war, it is not practicable to retain cases sufficiently long for one to commence any reconstructive procedures, and our operative procedures were therefore more in the nature of first aid. This does not sound very formidable or extensive, but, as in most other branches of surgery, the primary operation is an all-important one, and the decisions taken at this time influence profoundly the subsequent course of the case. In general terms, we tried as far as we could to fix every patient so that first, he was fit to travel, and secondly, he would need nothing more done to him until the next stage of treatment was under consideration. This aim means a lot of elaborate work for the dental surgeons and mechanics. It means that a complete and definitive operation is aimed at in every case.

In dealing with facial injuries, the primary objective is an early and accurate position of the bony framework, and after achieving this, the closure of the soft tissues is a secondary problem. The cases for treatment can be divided into two main groups: those with massive loss of tissue, and those in whom there was little or no loss. In the case of the former, the object to be aimed at is early and complete healing so that reconstructive procedures can be started at the earliest possible date. This can be achieved by careful and conservative excision of the wounds, and careful closure of the mucous membrane to the skin wherever possible. Extensive raw areas due to large loss of skin can be covered by free grafts, which take well on an area which has been adequately excised, and where a reasonable degree of hæmostasis has been obtained. Where there has been massive loss of bone, no useful purpose is served by attempting to fix the few small fragments which remain.

It has been shown, and our experience confirmed this, that primary suture of facial and scalp wounds can be undertaken several days after they have been sustained. Tetanus and gas gangrene infections are almost unknown in connection with wounds of this type, and so it is justifiable, even with dirty battle casualties, to consider primary closure, when one would not contemplate it elsewhere. Conservative but adequate excision of all damaged and non-viable tissue, followed by careful and accurate suturing, is the way to ensure good results. Some small amount of tension is permissible, but it is futile to approximate tissues forcibly which normally are not adjacent or continuous. If the stitches do hold, the subsequent result is a considerable deformity which has to be corrected later, but more often, the stitches fail to hold, and cut out, producing further destruction, and ultimately more loss of living tissue. The vascularity of the face and scalp is well known, and it is probably one of the reasons for scarcity of anærobic infections. The enormous blood supply unquestionably helps to overcome infection, and results in early and satisfactory union, and it is possible, therefore, to remove stitches far earlier than elsewhere in the body. We used penicillin and sulphonamides freely, as routine for all cases, and one was constantly being surprised by the absence of infection and reaction in wounds which had been sutured late.

*Combined Maxillo-facial and Neurological Injuries.* We had a number of cases through our hands in which the facial injuries were either associated with, or in direct continuity with, intra-cranial wounds. One example of this type of injury, illustrated in Fig. 3, is the so-called central, or "dishface" injury. The increasing number of fast-moving vehicles and aeroplanes has resulted in this injury becoming common. The vehicle or aeroplane comes to an abrupt stop, and the patient's face comes into violent contact with the windscreen, dashboard, instrument panel, or some other unyielding surface, and it is found that the whole of the centre part of the face is displaced backwards, and is loose from its attachments to the skull. These high maxillary fractures may, and often do, involve the cribiform plates, and meningitis may follow. Many of these cases suffer from cerebro-spinal rhinorrhœa, which may, or may not, be clinically detectable.

This case (Fig. 3) is an example of this disaster. This man sustained an extensive, high fracture of the maxillary block and, within eighteen hours, exhibited rapidly progressing signs of meningitis. Cultures made from the cerebro-spinal fluid resulted in a vigorous growth of pneumo-cocci. The infection responded dramatically to penicillin, and in four days he had regained consciousness. Steps were then taken to stabilize the maxilla, and to prevent further damage to the area of the cribiform plates. Cerebro-spinal fluid continued to leak from the wound on the bridge of the nose, and as soon as he was considered fit to undergo the operation, a frontal flap was raised by Major J. M. Small, and a large graft of fascia lata was placed over the cribiform plates, to close and seal off the cerebro-spinal leakage. This procedure was immediately effective, and the patient made a very satisfactory recovery. All patients with high maxillary fracturing should be regarded as possible candidates for meningitis, and fracturing of the cribiform area should be excluded by testing the patient's sense of smell. As soon as the nose has been cleared from blood clot, and the patient has a reasonable airway through it, tests should be made to find out if there is anosmia. If the sense of smell is materially altered or absent, it is strongly suggestive that there has been fracturing and damage in this important area. Even high-class X-ray photography sometimes fails to reveal direct visual evidence of fractures, and such patients who are under suspicion should be seen by a neuro-surgeon.

Another type of compound maxillo-facial and neurological injury is illustrated in Fig. 4. Here, there is direct continuity between the face and brain by means of a large external wound. This soldier sustained, as can be seen in the photograph, a major injury, involving the left orbit and frontal lobes. There was extensive loss of skin, bone and dura. The brain wound was dealt with by Major J. M. Small, who carefully excised the wound, and all pieces of bone and damaged brain. At the end of the operation, there was a very large defect, and the problem to be dealt with was to obtain satisfactory cover for the exposed brain tissue. It was considered that a living flap of tissue, carrying its own blood supply, was the answer, and this flap was obtained by utilizing a huge scalp flap, rotating downwards and forwards to meet the tissues of the face below the orbit. Fig. 4B shows the appearance four days later, when the patient was intelligent and co-operative, and exhibiting no gross neurological signs. He was evacuated by air to England, where subsequent repair procedures have been continued by Professor Pomfret Kilner, the final plan being to replace the scalp flap to its normal site, when adequate skin cover for the damaged brain has been brought to the vicinity to re-constitute his forehead. This has been done by Professor Kilner, who has used a tantalum plate to restore the bony contour of the forehead.

It has not been possible in the time and space allowed, to give more than just a sketchy outline of the types of cases we had to deal with, and only a superficial survey of the methods employed in treating them. I hope I have said enough to make it plain that work of this kind is only possible by teams, each member of which should be specially trained. I have no doubt at all that high-class work in complicated specialities can only be done by teams, and I think that most of us who were in the Services are convinced that the policy of providing specialist units was fully justified by the results.

## Figures and Tables

**Fig. 1. f1:**
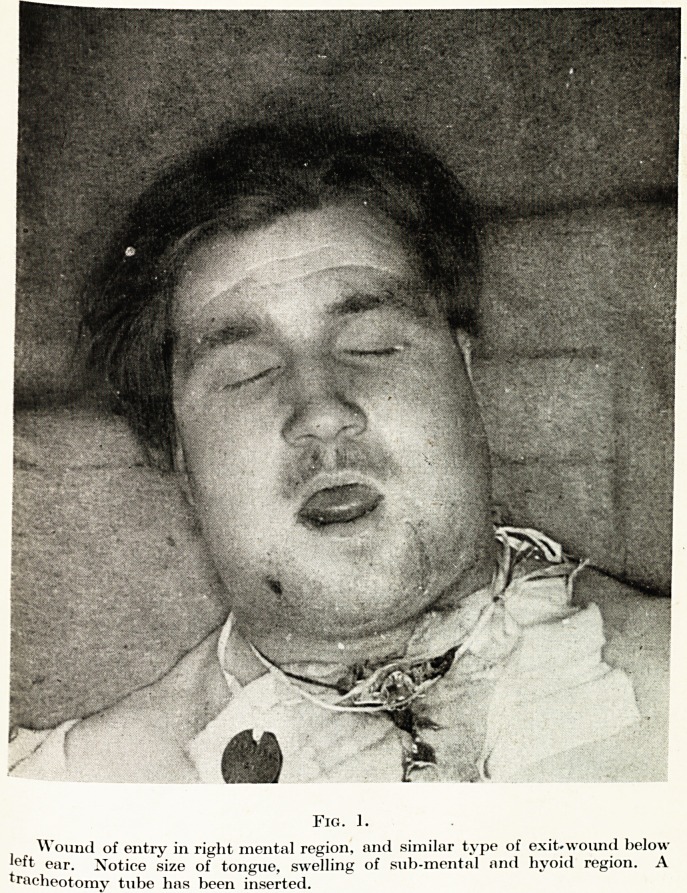


**Fig. 2a. f2:**
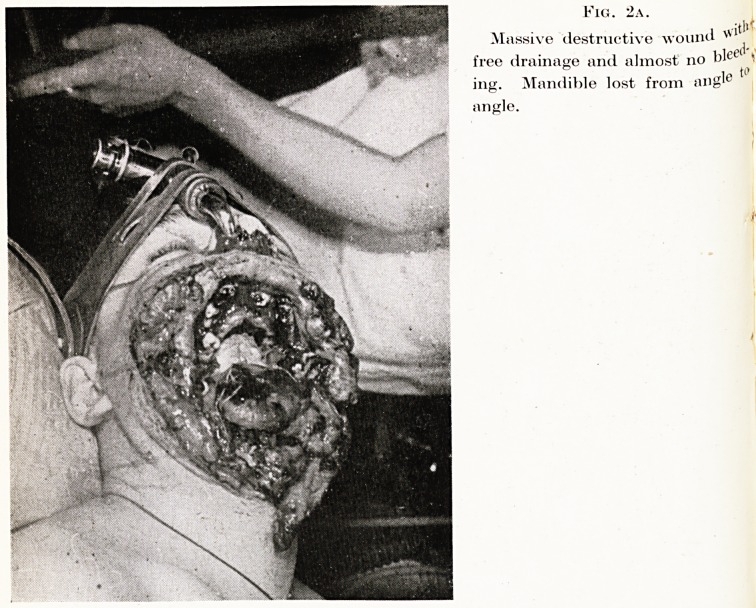


**Fig. 2b. f3:**
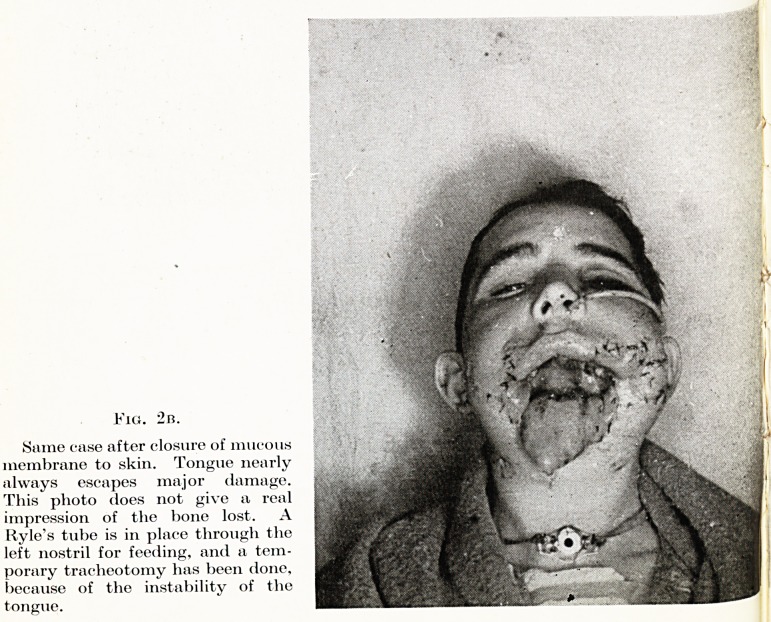


**Fig. 3. f4:**
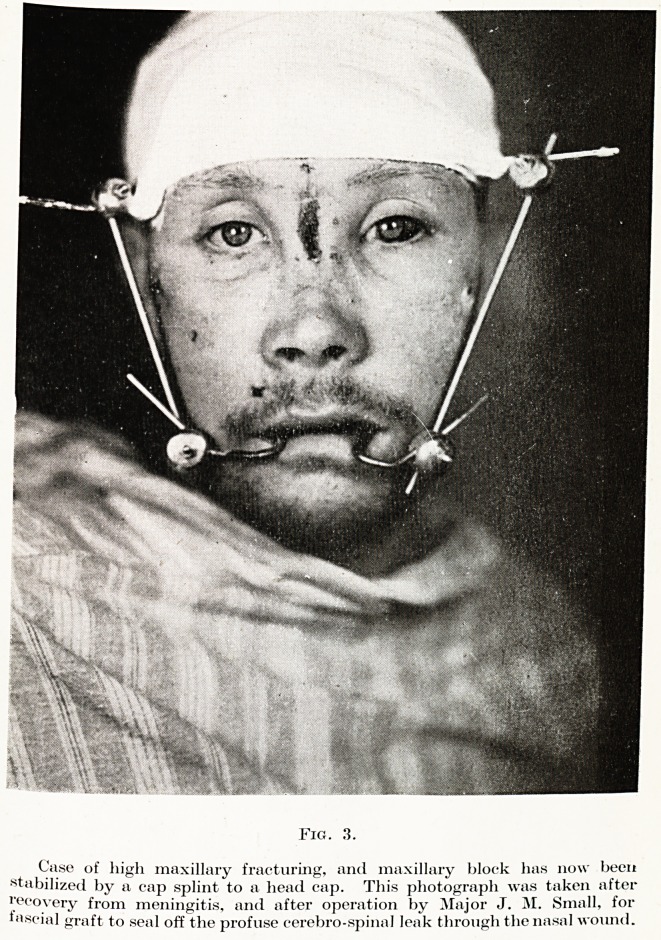


**Fig. 4a. f5:**
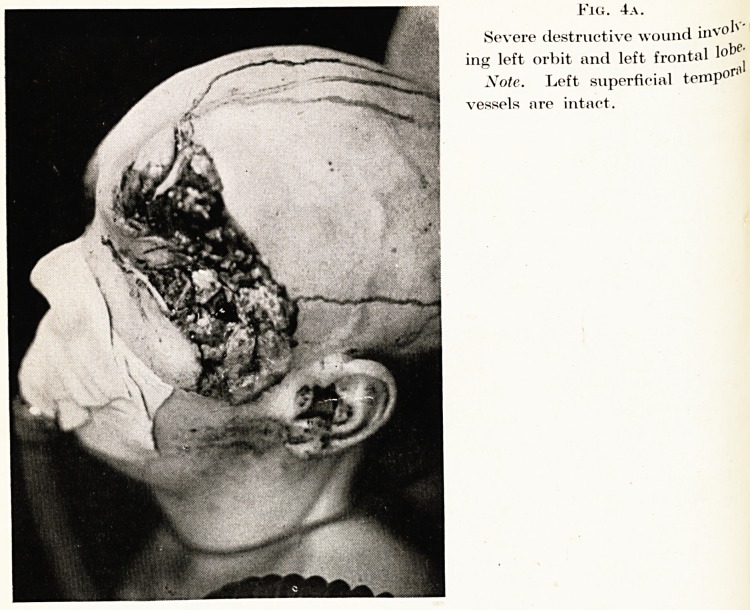


**Fig. 4b. f6:**