# Correction

**Published:** 1947

**Authors:** 


### CORRECTION.

The date on which Mr. FitzGibbon read his paper on "**War Injuries of the Face and Jaws,**" was October 9th, 1946; *not* 1947 as stated on page 61 of the Autumn Number.

